# Comparative analysis of anthelmintic treatments: impact on liver biomarkers and clinical recovery in sheep with fasciolosis

**DOI:** 10.3389/fvets.2025.1485568

**Published:** 2025-02-20

**Authors:** Tameru Gedefaw, Atsede Solomon Mebratu, Shimels Dagnachew, Melkie Dagnaw Fenta

**Affiliations:** ^1^Department of Veterinary Clinical Medicine, University of Gondar, Gondar, Ethiopia; ^2^Department of Veterinary Pharmacy, University of Gondar, Gondar, Ethiopia; ^3^Department of Veterinary Parasitology, University of Gondar, Gondar, Ethiopia

**Keywords:** anthelmintics, biochemical parameters, clinical recovery, effectiveness, fasciolosis, sheep, West Dembiya

## Abstract

**Background:**

Liver fluke infections (fasciolosis) in sheep in Ethiopia result in significant economic losses, recently estimated at around $3,700 (185,232 ETH Birr) per year. Despite the widely use of Triclabendazole (TCBZ), Tetraclozan (TETRA), and Albendazole (ALBE) for treating fasciolosis, their effectiveness remains a significant concern. This study aimed to compare the efficacy of TCBZ, TETRA, and ALBE in treating ovine fasciolosis, with a focus on their effects on both the parasitic infection and associated biochemical parameters. Given the substantial economic burden of liver fluke in Ethiopia, identifying the most effective treatment options is essential to reducing both the health impact on livestock and the economic losses to farmers.

**Methods:**

A field trial was conducted from May to November 2023 on 45 naturally infected sheep, divided into three groups: Group I received TCBZ, Group II TETRA, and Group III ALBE, with TCBZ as the positive control. Fecal and serum samples were collected on days 0, 7, 14, and 21 post-treatment. A fecal egg count reduction test (FECRT) and biochemical analysis were performed.

**Results:**

In our study, TCBZ was the most effective anthelmintic (97.8%), followed by TETRA (96.6%), and ALBE (84%). Biochemical parameters, particularly liver enzymes (AST, ALT, ALP, GGT) and protein levels, showed significant improvement across all groups over 21 days (*p* < 0.05), with enzyme levels normalizing by day 21 and protein levels by days 14-21. Albendazole significantly (*p* < 0.05) outperformed Tetraclozan and Triclabendazole in both biochemical parameters and eggs per gram count (EPG), with no significant difference between Tetraclozan and Triclabendazole (*p* > 0.05). Albendazole proved most effective for liver recovery and normalization of biochemical markers over the treatment period (*p* = 0.00). Among 15 Fasciola-infected animals, baseline signs included diarrhea (53%), pale mucous membranes (100%), bottle jaw (60%), and depression (80%). Post-Tetraclozan treatment, all symptoms reduced significantly over 21 days (*p* < 0.05). In the Albendazole group, symptoms decreased progressively, with diarrhoea, pale mucous membranes, bottle jaw, and depression notably reduced by days 7, 14, and 21.

**Conclusion:**

TCBZ and TETRA were highly effective against ovine fasciolosis, with TETRA recommended if TCBZ is unavailable. Biochemical parameters are key biomarkers for liver damage and selecting effective anthelmintic drugs.

## 1 Introduction

Livestock are a primary source of income and food security worldwide. Ethiopia has the largest livestock population in Africa, with 65 million cattle, 40 million sheep, and 51 million goats (1). Sheep are the most common livestock in Ethiopia, contributing up to 63% of all monetary income and 23% of the total value of food products from livestock. Due to the relatively low cost of breeding stock, high productivity rates, and financial gain potential, sheep production is a popular agricultural endeavor in Ethiopia (2). However, infectious and noninfectious diseases have become major constraints on sheep productivity, with helminthic diseases, particularly fasciolosis, being prominent parasitic threats (3). Several trematodes affecting livestock and cattle in Ethiopia are associated with fasciolosis, particularly *Fasciola hepatica* (*F. hepatic*a) and *Fasciola gigantica (F. gigantica*). These *Fasciola* species, transmitted by *Lymnaea truncatula* and *L. natalensis* snails, are clinically associated with symptoms such as bottle jaw, anemia, emaciation, diarrhea, and potbelly (4, 5). The highlands of Ethiopia, especially the Lake Tana area in West Dembiya, are particularly susceptible to fasciolosis due to swampy conditions that support the spread of snail hosts. This region is therefore ideal for studying the impact of the disease on livestock health (3, 4).

Current control methods rely heavily on anthelmintic treatments, such as Triclabendazole, Albendazole, and Tetraclozan, yet concerns about drug resistance and poor-quality veterinary drugs complicate treatment outcomes (6, 7). Despite their widespread use, there is limited research on the effectiveness of these treatments in Ethiopia, particularly in areas with unique ecological conditions like West Dembiya, where the snail hosts thrive.

This study aims to address this gap by evaluating the efficacy of these anthelmintics in treating ovine fasciolosis, focusing on clinical recovery, fecal egg count reduction tests, and biochemical parameters. By focusing on a high-risk, ecologically sensitive area, this research seeks to provide critical insights into treatment effectiveness and potential resistance, ultimately improving fasciolosis control in Ethiopia. The infection leads to inflammation and damage in the liver, which can result in elevated levels of liver enzymes, including alanine aminotransferase (ALT), aspartate aminotransferase (AST), and alkaline phosphatase (ALP), commonly used as diagnostic biomarkers for liver dysfunction. Triclabendazole, Tetraclozan, and Albendazole are widely used anthelmintics for treating *Fasciola* infections; however, a comprehensive comparison of their effectiveness in liver enzyme levels and restoring liver function in sheep with Fasciolosis has not been thoroughly investigated. Triclabendazole, Tetraclozan, and Albendazole are commonly used anthelmintics for *Fasciola* infection, yet their comparative effects on liver enzyme levels and liver function in ovine fasciolosis are insufficiently explored. Therefore, this study aimed to compare the effect of Triclabendazole (TCBZ), Tetraclozan (TETRA), and Albendazole (ALBE) in treating ovine fasciolosis, focusing on their impact on the parasitic infection, clinical recovery and associated biochemical parameters, particularly liver enzymes.

## 2 Materials and methods

### 2.1 Study area

**Figure 1:**
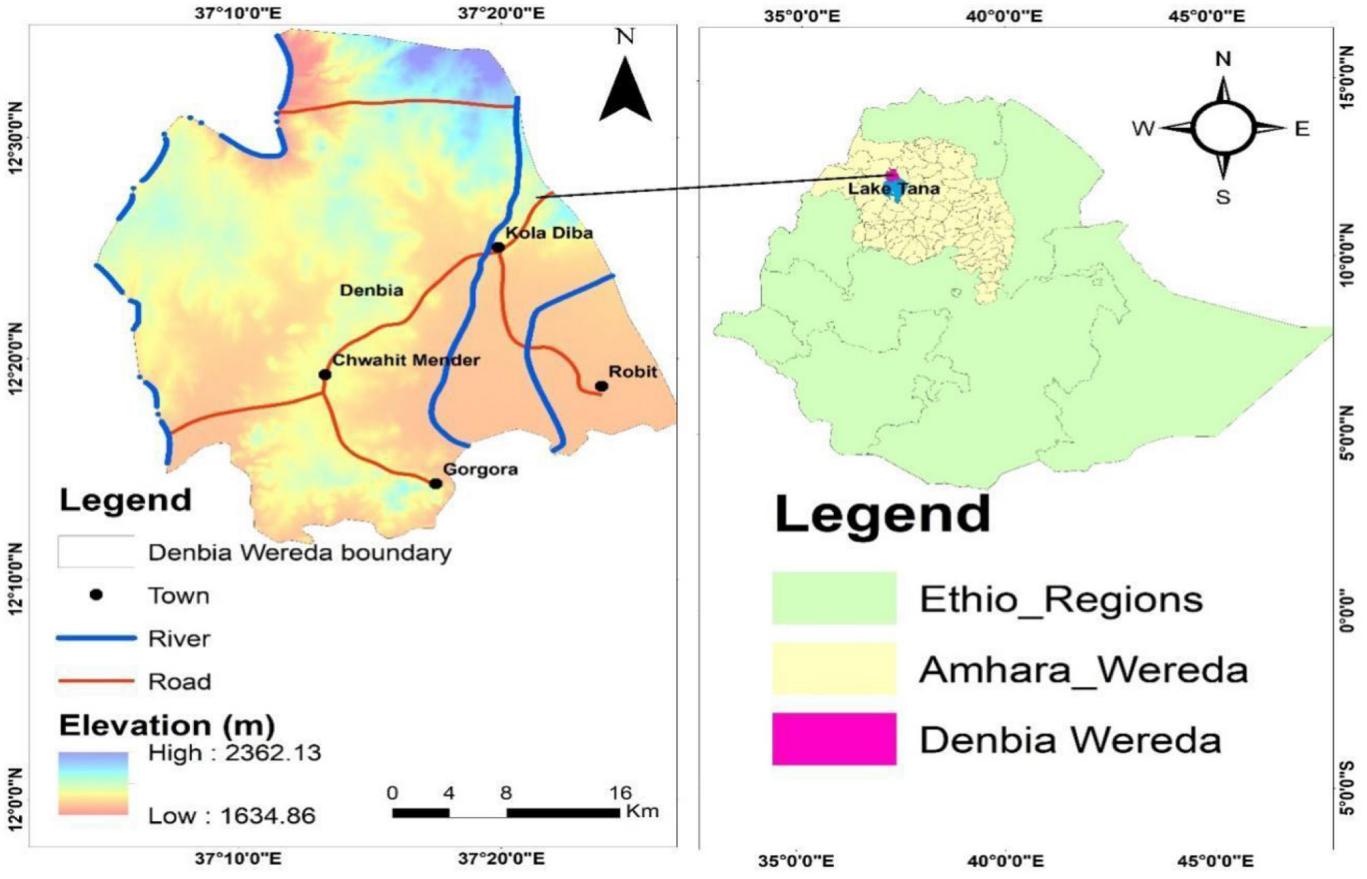
Study area (ARC GIS).

This field trial was conducted in the West Dembiya district, located within the Central Gondar Zone of the Amhara Region, Northwestern Ethiopia (Figure 1). The district is bordered by East Dembiya to the east, Chilga to the north, Alefa to the west, and Lake Tana to the south. It is about 765 km from Addis Ababa, the capital of Ethiopia, and 45 km from Gondar town (8). The area's elevation ranges from 1,500 to 2,600 m above sea level, with a topography characterized by Woynadega (Midland) agroecology, including floodplains, flat terrain, and swampy areas ideal conditions for the development of the intermediate snail hosts of *Fasciola*, the parasites responsible for fasciolosis.

The district was selected due to its high prevalence of fasciolosis, which is linked to the local topography and climate. The swampy floodplains and proximity to Lake Tana provide optimal breeding grounds for Lymnae species snails, which are essential for the *Fasciola* parasite's life cycle. These areas experience increased humidity, moderate temperatures, and seasonal rains, creating an environment that supports the proliferation of these snails and increases the transmission risk of fasciolosis. Additionally, the region's elevation (ranging from 1,500 to 2,600 meters) places it in the Woynadega agroecological zone, which is prone to seasonal flooding and waterlogging, further enhancing the survival and spread of the parasite. This ecological backdrop contributes to the endemic nature of fasciolosis in the area, making it an important location to evaluate the effectiveness of treatments, such as Triclabendazole, Albendazole, and Tetraclozan.

Furthermore, the district's climate can also impact the efficacy of anthelmintic drugs. Rainy seasons may alter the pharmacokinetics and bioavailability of the drugs, while humid conditions may favor the persistence of drug residues in the environment, possibly influencing treatment outcomes. With a dense livestock population and favorable conditions for Fasciola transmission, West Dembiya presents an ideal setting to assess the efficacy of anthelmintics under local environmental conditions. The study also aims to address the issue of substandard veterinary drugs and their effect on the effectiveness of fasciolosis treatments.

### 2.2 Study design and period

A field experimental trial was conducted from May to November 2023 to evaluate the effectiveness of Albendazole, Tetraclozan, and Triclabendazole in treating fasciolosis in naturally infected sheep. To assess the effectiveness of each treatment in reducing parasitic load, three key assessments were conducted: (1) a fecal egg count reduction test, (2) an analysis of liver biomarkers including AST, ALT, ALP, GGT, total protein (Tp), and albumin (Alb) for monitoring liver function and treatment impact, and (3) clinical evaluations to measure changes in symptoms and overall health. Results were analyzed to determine the comparative effectiveness of each treatment in controlling fasciolosis and its associated liver dysfunction.

### 2.3 Ethical consideration

The current trial adhered to ethical standards for the use of animals in research. Ethical approval was obtained from the College of Veterinary Medicine and Animal Science Ethical Committee at the University of Gondar (Reference: CVMASc/UoG/RERC/21/04/2023). The research followed internationally recognized guidelines on the humane treatment of animals in experimental studies. Before any animals were included in the study, informed verbal consent was obtained from the owners of the sheep. They were fully informed about the nature of the research, the procedures involved, and the potential impacts on their animals. The consent process ensured that owners were aware of their rights to withdraw animals from the study at any point. The study was conducted with the utmost consideration for animal welfare, and every effort was made to minimize any potential discomfort or harm to the animals throughout the trial.

### 2.4 Study population and selection procedures

Sheep were selected from smallholder herds in the Amhara region, chosen for their high prevalence of fasciolosis and uniform environmental conditions conducive to the disease. Several potential biases and confounding factors could have influenced the outcomes of this study. To mitigate this, sheep were in adult age category and all same sex (male). Breed differences were also considered, as certain breeds may show varying levels of resistance to infection. Only common breeds (local) in the study area were included. Additionally, environmental and seasonal variations, such as changes in grazing conditions, could influence animal health. To minimize these effects, the study was conducted over a specific time period (one season) and in a similar area using the same watering system (common watering). Only grazing animals in small stakeholder groups were included. A total of 75 sheep showing clinical signs of fasciolosis (depression, pale mucous membranes, bottle jaw, and diarrhea) were initially screened, and laboratory testing confirmed *F. hepatica* infection in 45 sheep, which were then included in the study. Only animals from extensive grazing systems and those untreated with anthelmintics in the past month were eligible, ensuring sample representativeness. The 45 fasciolosis-positive sheep (15 per group) were randomly assigned to three treatment groups, each receiving a different anthelmintic (Triclabendazole, Tetraclozan, or Albendazole) at manufacturer-recommended dosages. A random number generator was used to assign each sheep a unique ID, ensuring even distribution across the groups and minimizing confounding variables to maintain similar group characteristics.

This sample size was chosen to balance logistical feasibility with statistical power, aiming to provide representative and generalizable insights into anthelmintic efficacy in the region.

#### 2.4.1 Anthelmintics used for this trial

All the anthelmintics used for this field trial were approved by the Ethiopian Agricultural Authority (EAA). Table 1 shows the recommended dosage regimens for the three anthelmintic drugs used to treat fasciola-infected sheep. Each drug's commercial name, dose, manufacturer, and country of origin are detailed to ensure consistency and adherence to therapeutic guidelines. Each sheep in the Triclabendazole group received a dose of 10 mg/kg body weight, while Tetraclozan and Albendazole were administered at 10 and 7.5 mg/kg, respectively. Doses were given orally using boiling gun to ensure accuracy, with each sheep's body weight measured before dosing for precise calculation. Treatment was administered in a single dose on Day 0 to standardize drug exposure across groups, enhancing the reliability of efficacy comparisons. Sheep weights varied, with a minimum of 25 kg and a maximum of 45 kg: 11 sheep weighed 25 kg, 10 weighed 30 kg, nine weighed 35 kg, 11 weighed 40 kg, and foue weighed 45 kg. Based on these weights, the specific dose for each sheep was calculated accurately to align with the recommended body-weight based dosage for each anthelmintic. Table 2 provides the dosage of each drug based on the sheep's body weight.

**Table 1:** Anthelmintics, manufacturer, and dose in the trial against fasciolosis.

**Group name**	**Trade name**	**Generic name**	**Country**	**Dose/kg**	**M**	**RA**	**Mgf. date**	**EXP. date**
TCBZ	Fasinix	Triclabendazole	India	10 mg/kg	Ashish Life science	Oral	9/2022	9/2025
TETRA	Tetracozan	combined tetramisole with oxyclozanid	India	10 mg/kg	Ashish Life science	Oral	4/2022	4/2025
ALBE	Alben	Albendazole	China	7.5 mg/kg	Hebei Yuanzheng	Oral	6/2022	8/2025

TCBZ, Triclabendazole; TETRA, Tetraclozan; ALBE, Albendazole; RA, Route of administration; N, number of sheep included in each group; M, Manufacturer.

**Table 2:** Doses of each drug according to the sheep's body weight.

**Sheep weight/kg**	**Triclabendazole dose (10 mg/kg)**	**Tetraclozan dose (10 mg/kg)**	**Albendazole dose (7.5 mg/kg)**	**Number of sheep**
25	250 mg	250 mg	187.5 mg	11
30	300 mg	300 mg	225 mg	10
35	350 mg	350 mg	262.5 mg	9
40	400 mg	400 mg	300 mg	11
45	450 mg	450 mg	337.5 mg	4

#### 2.4.2 Sample size determination and justification

The sample size of 75 sheep, with 45 sheep included in the final analysis (those confirmed positive for *F. hepatica*), was determined based on the observed clinical signs in the study area and the resources available for the study. Although power calculations were not conducted due to practical constraints (e.g., resource limitations and the availability of infected animals), the sample size (15 in each group) was deemed sufficient to detect meaningful differences in the effectiveness of the anthelmintic treatments. The inclusion of 45 confirmed positive animals ensures that the study population is adequately represented and focused on animals actively infected with *F. hepatica*. Moreover, the study design involved purposive sampling of animals based on clinical signs, providing a robust basis for comparing treatment efficacy. The relatively large proportion of infected sheep [45/75 ( 60%) of the total sample] strengthens the validity of the findings. This 60% of the sampled sheep being infected, the high infection rate improves the study's power to detect treatment effects, even with a smaller sample size. This proportion provides a concentrated focus on infected animals, enhancing the likelihood of observing clinically relevant changes and biochemical parameters in response to treatment. For outcomes such as clinical recovery and biochemical parameters, smaller sample sizes can effectively detect differences, especially when the expected effect size is substantial. Since the study focuses on parameters that are likely to show significant responses to treatment, a sample size of 15 animals per group is considered sufficient to yield meaningful and reliable results.

### 2.5 Field experimental trial procedures

After the owners' permission to use their animals for this experimental study was obtained, all the sample bottles were marked with specific IDs, owner names, and collection dates. In this study, the effectiveness of three anthelmintic treatments Triclabendazole (TCBZ), Tetraclozan (TETRA), and Albendazole (ALBE) was evaluated using three primary experimental methods: the fecal egg count reduction test (FECRT), clinical recovery and liver biomarker analysis. Fecal samples were collected from each sheep at four-time points (0, 7, 14, and 21 days post-treatment) and analyzed using a modified McMaster technique to quantify the trematode eggs per gram (EPG) of feces. In this study, the 45 fasciolosis-positive sheep were randomly assigned to three treatment groups (Albendazole, Tetraclozan, and Triclabendazole) using a completely control randomization method. Each sheep was assigned a unique number, and the random number generator was used to allocate them evenly across the three groups. By employing a random allocation method, we ensured that the treatment groups were as similar as possible in terms of their characteristics, helping to isolate the effects of the treatments from other confounding variables. The FECRT was then calculated by comparing the pre-treatment egg count with the post-treatment counts to assess the reduction in fecal egg counts and determine the anthelmintic efficacy, with a reduction of ≥95% indicating effective treatment. Samples before treatment (Zero-day samples) were considered the negative control, and the Triclabendazole Group was considered the positive control. Additionally, blood samples were collected from the jugular vein at the same time points to assess liver function by measuring biomarkers such as AST, ALT, ALP, GGT, total protein (TP), and albumin (Alb). These biomarkers were analyzed using a fully automated biochemical analyzer to evaluate liver damage or dysfunction caused by *F. hepatica* infection. These combined methods allowed for a comprehensive evaluation of the efficacy of the three anthelmintic treatments in managing fasciolosis and their impact on liver function, ensuring the reliability of the study's findings.

#### 2.5.1 Control groups and justifications

In this study, two control groups were used to ensure the validity of the treatment comparisons: a positive control and a negative control. Positive Control (Triclabendazole Group-TCBZ): the group treated with Triclabendazole (TCBZ) was designated the positive control. TCBZ is a widely used and well established treatment for fasciolosis, and its inclusion in the study allowed for comparison of the experimental treatments (Tetraclozan and Albendazole) to a standard, effective treatment. This group provided a benchmark for assessing the efficacy of the other treatments. Additionally, this approach aligned with ethical standards in animal research by ensuring that at least one group received an effective treatment, which likely alleviated suffering associated with untreated fasciolosis and improved animal welfare outcomes.

Negative Control (Zero-Day Samples): the zero-day samples (taken before treatment) were the negative control. These baseline samples were used to assess the pre-treatment condition of the animals and provided a reference point for evaluating any changes in clinical signs or biochemical parameters resulting from the treatments. The zero-day samples were compared with post-treatment data to evaluate the effectiveness of each treatment. The positive control group (TCBZ) allowed us to assess the expected improvement in clinical signs and biochemical markers based on an established, effective treatment. By comparing the results of Tetraclozan (TETRA) and Albendazole (ALBE) groups with the TCBZ group, we could determine whether these treatments showed similar, better, or worse results than TCBZ. The negative control (zero-day samples) served to identify any changes in the animals' conditions before the start of treatment, ensuring that observed effects in the treated groups were due to the treatment and not to natural recovery or external factors. The changes in clinical signs and biochemical markers (e.g., liver enzymes, protein levels) were compared between the treatment groups (TETRA, ALBE, and TCBZ) and the control groups (zero-day samples and TCBZ). This comparison helped assess the relative efficacy of the treatments and allowed for statistical analysis of the differences between groups.

#### 2.5.2 Detailed clinical data

The study collected comprehensive clinical data to evaluate the health status and treatment response of the sheep across different clinical parameters. Key indicators, including diarrhea, pale mucous membranes (M/m), bottle jaw, and signs of depression, were documented for each group before and after treatment. Data collection was conducted after obtaining permission from the sheep owners and verifying the animals' deworming history to ensure accurate baseline information.

In Group 1, 60% of the sheep exhibited diarrhea, while 53% in Group 2 and 73% in Group 3 showed similar symptoms. Pale mucous membranes, a consistent indicator of infection, were observed in 100% of the sheep across all groups. The prevalence of bottle jaw was 33% in Group 1, 60% in Group 2, and 47% in Group 3, reflecting variations in clinical severity. Depression, indicative of the disease's impact on overall health, was observed in 80% of sheep in both Group 1 and Group 2, and in 73% of Group 3.

All clinical signs were systematically recorded in a standardized record book to ensure consistency and reliability of data collection. Each parameter was assessed by trained personnel using a standardized clinical assessment form at predetermined intervals, capturing any changes in the sheep's health status throughout the treatment period. This meticulous approach to data collection provided a robust basis for analyzing the effectiveness of the anthelmintic treatments, yielding reliable and clinically meaningful insights into health improvements in each treatment group. Clinical observations of the sheep, including signs like diarrhea, pale mucous membranes, bottle jaw, and depression, were also recorded at each time point to track the overall clinical recovery (Table 3).

**Table 3:** Observed clinical presentation before treatment for each Group.

**Clinical presentation**	**Group-1**	**Group II**	**Group-3**
Diarrhea	9 (60%)	8 (53%)	11 (73%)
Pale M/m	15 (100%)	15 (100%)	15 (100%)
Bottle jaw	5 (33%)	9 (60%)	7 (47%)
Depression	12 (80%)	12 (80%)	11 (73%)

### 2.6 Sample collection, storage and laboratory procedures

Fecal samples were collected directly from the rectum and blood from the jugular vein via blood clotting activator-coated test tubes on days 0, 7, 14, and 21. The fecal samples were then placed into universal sampling bottles filled with 10% formalin. The samples were subsequently stored in an ice box and transported to the Parasitology and Pathology Laboratory at the University of Gondar. To maintain their integrity, the fecal and serum samples were frozen in a refrigerator at temperatures of 4 and −21°C, respectively. Fecal examination and biochemical analysis were used for laboratory investigations to determine the fecal egg count per gram of feces and the level of biochemical parameters (liver biomarkers). To maintain egg integrity and prevent changes to larvae, 10% formalin was used for preservation, as it helps to prevent egg development and distortions, especially if there is a delay in processing (3–4 days). While storing fecal samples at 4°C can slow down egg development, it is not sufficient for long-term preservation, as eggs may still undergo changes that could affect the accuracy of counts. Therefore, formalin was chosen as a more reliable preservative to maintain the integrity of trematode eggs over time. Although formalin can sometimes affect the accuracy of fecal egg counts by distorting egg morphology or reducing egg viability, it is crucial for preventing larvae development and preserving egg stability.

The study was conducted in full compliance with ethical guidelines for animal research. Participation in the trial, including the collection of blood and fecal samples, was carried out with the informed consent of the sheep owners. The study was approved by the University of Gondar's Institutional Animal Ethics Committee, which ensures that all procedures involving live animals are performed with the highest standards of care and ethical responsibility. Efforts were made to minimize animal discomfort and stress during the sample collection process, with all procedures performed by trained personnel. Blood samples were collected using sterile techniques from the jugular vein, and fecal samples were obtained in a manner that did not harm the animals. Additionally, the study adhered to the principles of the 3Rs (Replacement, Reduction, and Refinement) to ensure that the number of animals used was minimized while still maintaining the statistical power of the trial.

*Liver biomarkers:* it includes liver enzymes (AST, ALT, ALP, GGT) and protiens (Tp, and Alb).

#### 2.6.1 Fecal sample examination

Fecal samples used to identify trematode eggs were collected at 0, 7, 14, and 21 days of treatment. The presence of trematode eggs was determined qualitatively via the sedimentation technique. Approximately 3 g of feces were weighed and placed into a container, followed by the addition of 42 ml of tap water. The mixture was stirred thoroughly with a stirring device, such as a fork or tongue blade. The fecal suspension was then filtered through a tea strainer or a double layer of cheesecloth into a second container. The filtered material was poured into a test tube, and the samples were allowed to stand for 5 min to let the sediment settle. The supernatant was carefully removed by pipetting or decanting. After removing the supernatant, the sediment was transferred to a petri dish, and a drop of methylene blue (1%) was added to enhance visibility. The methylene blue stained the background for improved contrast, facilitating examination under a dissecting microscope. The sediment was then resuspended in 5 ml of water and allowed to sit for another 5 min. The supernatant was discarded again with care, and the sediment was examined under a microscope at 10 ×, objective magnification. *Fasciola* eggs were identified by their large size, operculated shape, and yellow-brown coloration, often requiring comparison with standard identification keys for confirmation (9).

#### 2.6.2 Fecal egg count reduction test

A quantitative analysis of the fecal samples was also conducted to determine the degree of infestation of fasciolosis via the Fecal Egg Count Reduction Test (FECRT) (10). To determine the number of EPGs modified McMaster technique with Zn_2_SO_4_ solution was used. A single chamber of the McMaster slide was filled with 0.15 ml of sample and examined under a light microscope at 10 × magnification to determine. The fecal Egg Count Reduction Testwas used to evaluate the effectiveness of anthelmintic drugs in sheep. The reduction in FEC from pre-treatment to post-treatment sampling was calculated via the formula originally devised for helminthic parasites, which compares the Group arithmetic mean FEC at pre-treatment (FEC_o_) with the Group arithmetic mean FEC at post-treatment sampling (FEC_At_). The percentage of anthelmintic effectiveness was determined via the equation FECR (%) = (FEC_Bo_-FEC_At_) × 100/FEC_Bo_ (10).

*A*_*t*_ = Arithmetric mean EPG of each group on days 7, 14, 21.
*B*_*O*_ = Arithmetric mean EPG of each group on day 0

The effectiveness of the drugs was tested according to the World Association for the Advancement of Veterinary Parasitology (WAAVP) recommendations for the detection of anthelmintic resistance in ruminants, horses, and swine (11).

#### 2.6.3 Biochemical analysis procedure

In the study, biochemical analysis was conducted on all 45 sheep included in the trial, as these animals were confirmed to be naturally infected with *F. hepatica* and received one of the three anthelmintic treatments. Blood samples for biochemical analysis were collected at four time points (pre-treatment on day 0, and then at 7, 14, and 21 days post-treatment) from each of the 45 sheep. These blood samples were analyzed for liver biomarkers, including AST, ALT, ALP, GGT, total protein (TP), and albumin (Alb). For the biochemical examination, about five milliliters of blood were drawn from the jugular vein of a single animal and collected into separate serum tubes containing a cold activator gel. The blood was allowed to sit undisturbed in a slanted position for 3–4 h to allow clot formation and serum separation. The serum was then transferred to 2 ml Eppendorf tubes and stored in a deep freezer at −21°C in the laboratory (12).

All the serum samples were nonhemolyzed, and the sample volume, along with the reagents, was measured using a microliter (μl) pipette. The cuvettes (sample solution holders) were thoroughly cleaned, and different pipettes were used for each sample to prevent cross-contamination. The correct volume of each sample was carefully loaded into the cuvettes, along with a control solution (matched in volume and cuvette type to the sample being analyzed). To ensure accuracy and reliability in biochemical measurements, we implemented strict quality control measures during sample processing. Pipettes used in this study were calibrated regularly following standard laboratory protocols to verify accurate volume delivery. Calibration checks were conducted at the beginning of the study and periodically throughout the data collection process, adhering to manufacturer specifications to minimize the risk of volumetric error. To further prevent cross-contamination between samples, dedicated pipettes and cuvettes were used for each sample. After each measurement, pipettes and cuvettes were thoroughly cleaned and inspected to confirm they were free of residual material. These procedures were strictly adhered to in all sample preparations to maintain the integrity of biochemical measurements. This approach reduces potential contamination and measurement error, enhancing the reliability of the study's findings. The serum samples and reagents were then processed using a fully automated chemical analyzer, the Beckman Coulter DXC 700 AU (Beckman Coulter, Brea, CA, USA). The product was measured photometrically. Serum levels of aspartate aminotransferase (AST), alanine aminotransferase (ALT), gamma-glutamyl transferase (GGT), and alkaline phosphatase (ALP) were measured in IU/L, while total protein and albumin levels were measured in g/dl (13, 14). To ensure methodological accuracy and reliability, validation and calibration procedures were performed on all techniques and equipment used in this study. The analytical methods for biochemical assessments were validated by conducting preliminary tests to confirm that they produced accurate and reproducible results under study conditions. These validation tests involved running standard solutions and reference samples to verify the sensitivity, specificity, and accuracy of the assays. Additionally, equipment such as pipettes, spectrophotometers, and analyzers was calibrated regularly following manufacturer guidelines. Calibration was conducted at the beginning of the study and periodically throughout the experimental period to ensure consistent performance. By incorporating these validation and calibration procedures, we aimed to enhance the precision and reliability of the techniques used, thereby ensuring that the measurements accurately reflected the study parameters. The normal reference ranges of each biochemical parameter are listed in Table 4.

**Table 4:** Reference ranges of the serum biochemical parameters.

**Biochemical parameters**	**Unit**	**Sheep**	**Goat**	**Cow**
AST	U/L	49–123	66–230	60–125
ALT	U/L	15–44	15–52	6.9–35
ALP	U/L	27–156	61–283	18–153
GGT	U/L	20–44	20–50	6–17.4
TP	g/dl	5.9–7.8	6.1–7.5	6.7–7.5
Alb	g/dl	2.7–3.7	2.3–4	2.5–3.8

Source: Babják et al. (15).

ALP, alkaline phosphatase; GGT, gamma glutamyltransferase, AST, aspartate aminotransferase, ALT, alanine aminotransferase, ALP, alkaline phosphatase, Tp, total protein and Alb, albumin.

### 2.7 Data management and analysis

All the data were recorded in a predesigned format and entered into a computer via a Microsoft Excel spreadsheet. The data were analyzed using SPSS software. Descriptive statistics (frequencies, percentages, means, and standard deviations) were used to summarize clinical presentations and continuous variables such as liver biomarkers and fecal egg counts. To compare the effectiveness of the three treatments (TCBZ, TETRA, and ALBE), one-way ANOVA was conducted for continuous variables at different time points, with post-hoc pairwise comparisons (time with time interaction, time with treatment interaction, treatment with treatment interaction). Changes in liver biomarkers over time were analyzed using repeated measures ANOVA with a mixed-effects model. A *p*-value of < 0.05 was considered statistically significant. The FECRT (%) was calculated using 100 (1 – X_D_/X_0_), where X_D_ is the arithmetic mean of the post-treatment egg count at 7, 14 and 21 days and X_0_ is the arithmetic mean of the before-treatment Group at 0 days.

## 3 Results

### 3.1 Mean fecal egg count and reduction test

As shown in Table 5 and in supportive material-1, within Group I, the mean EPG (eggs per gram) count decreased from 1,727 to 80, 20, and 13 on days 7, 14, and 21, respectively. In Group II, the mean EPG count dropped from 1,807 to 140, 33, and 13 on days 7, 14, and 21, respectively. Similarly, in Group III, the mean EPG count decreased from 1,893 to 553, 240, and 107 on days 7, 14, and 21, respectively. The mean fecal egg count reduction tests (FECRTs) for Groups I, II, and III were 97.8%, 96.6%, and 84%, respectively.

**Table 5:** MFEC ± SD of fasciolosis in sheep before and after treatment.

**Anthelmintic groups**	**Mean EPG on days**				**EPG reduction at days (D) of treatment**		
	**D**_0_	**D**_7_	**D**_14_	**D**_21_	**D**_7_	**D**_14_	**D**_21_
TCBZ	1,726.7 ± 433.4	80.0 ± 77.5	20.0 ± 35.2	13.33 ± 35.20	95%	99%	99%
TETRA	1,806.7 ± 409.7	140.00 ± 63.3	33.33 ± 48.8	13.33 ± 35.20	91.7%	97.9%	99%
ALBE	1,893.3 ± 32.70	553.3 ± 272.2	240.0 ± 164	106.7 ± 70.40	70.4%	86.9%	94%

TCBZ, Triclabendazole; TETRA, Tetraclozan; ALBE, Albendazole; EPG, eggs per gram; MFEC ± SD, mean fecal egg count; standard deviation; D, day.

The fecal count reduction in Group I was 95%, 99% and 99%, the FECRT in Group II was 91.7%, 97.9%, and 99%, and the FECRT in Group III was 70.4%, 86.9% and 94% when FECR_7_ = (*D*_0_−*D*_7_)100÷*D*_0_, FER_14_ = (*D*_0_−*D*_14_)100÷*D*_0_ and FECR_21_ = (*D*_0_−*D*_21_)100÷*D*_0_ was used, respectively. As shown in Table 1, ALBE had a lower FECRT at 7, 14, and 21 days than did TCBZ and TETRA. The fecal egg count reduction test on day 21 was equal in treatment Groups I and II, whereas on days 7 and 14, TCBZ had the highest FECR compared with TETRA. The data indicate a significant reduction in the mean EPG count over time for all three groups, demonstrating the effectiveness of the treatments administered. Group I experienced the most substantial reduction, with the EPG count decreasing by 97.8%, followed closely by Group II with a 96.6% reduction. Group III showed a lower, though still notable, reduction of 84%. This suggests that the treatments in Groups I and II were more effective in reducing parasite load compared to the treatment in Group III. The consistent decrease in EPG counts over the 21-day period further supports the efficacy of the treatments across all groups.

#### 3.1.1 Comparative effects of treatment and time on eggs per gram count

**Figure 2:**
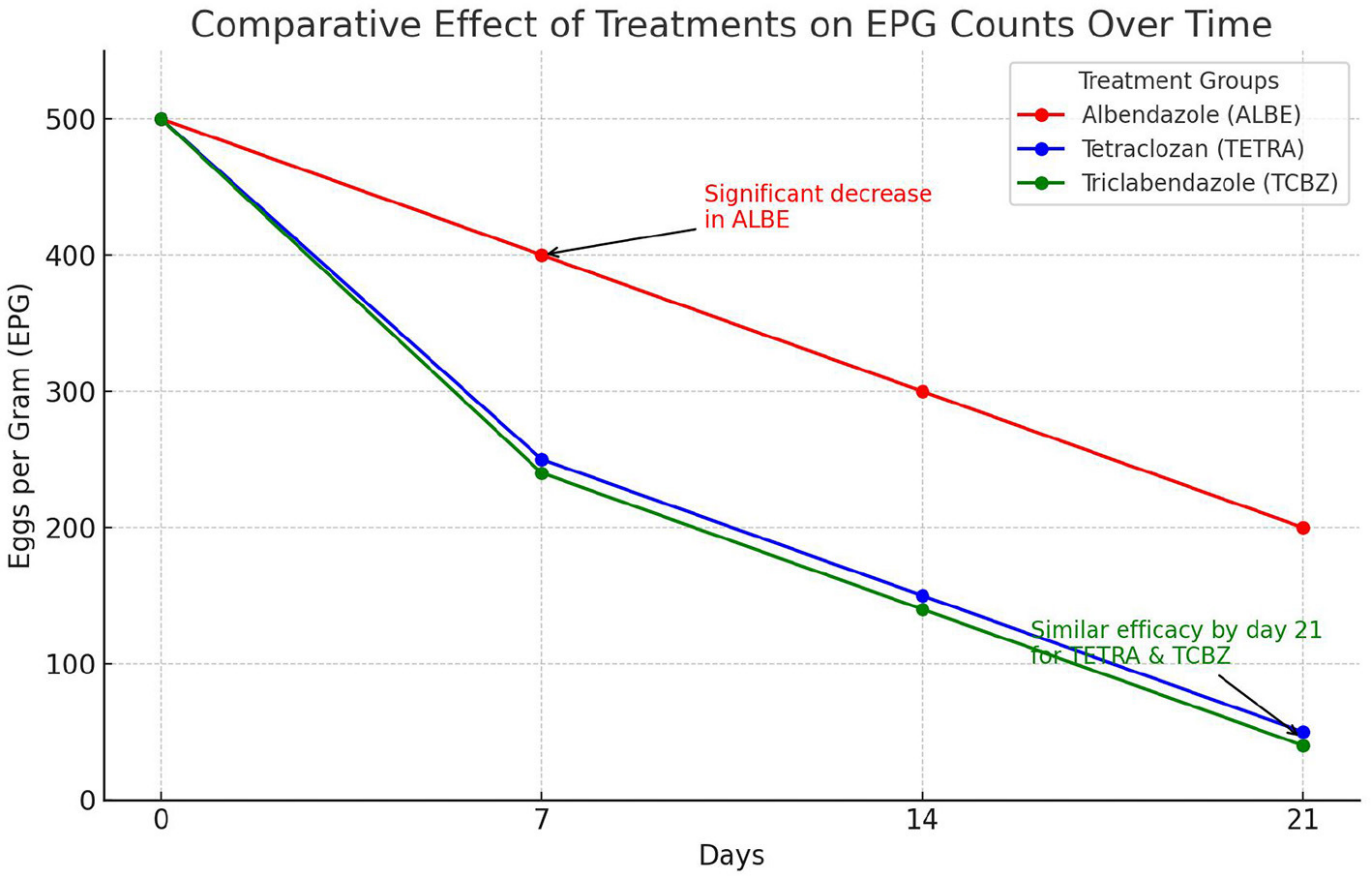
Mean number of eggs per gram with respect to time. This figure shows the comparative effects of three anthelmintic treatments Albendazole (ALBE), Tetraclozan (TETRA), and Triclabendazole (TCBZ) on eggs per gram (EPG) counts in sheep infected with liver fluke (fasciolosis) over a 21-day period. The y-axis represents the EPG count, indicating the parasite load, while the *x*-axis represents time intervals (0, 7, 14, and 21 days). The graph highlights a significant decrease in EPG counts across all treatments, with ALBE showing a less pronounced reduction compared to TETRA and TCBZ. By day 21, TETRA and TCBZ demonstrate similar effectiveness, as indicated by the convergence of their lines. This trend supports the efficacy of TETRA and TCBZ in reducing parasite load, with ALBE proving comparatively less effective over the study period.

On the basis of the observed mean EPG with respect to the time interval, the mean EPG decreased from day 0 to day 21. In the treatment rows in the TCBZ and TETRA Groups, the mean EPG had a greater reduction rate after 14 (3) and 21 (4) days of treatment, whereas in the treatment row in the ALBE Group, the mean EPG values did not decrease, similar to those in the TCBZ and TETRA Groups. The reduction rate of the MEPG was ranked as TCBZ >TETRA > ALBE with respect to time, but Figure 2 indicates that on day 21, TCBZ and TETRA overlapped. The ranking of effectiveness based on the reduction rate of the mean EPG was TCBZ > TETRA > ALBE. However, the overlap of TCBZ and TETRA reduction rates on day 21, as indicated in Figure 2, suggests that by the end of the observation period, both TCBZ and TETRA had achieved similar levels of effectiveness. This overlap may imply that, while TCBZ initially had a greater reduction rate, TETRA eventually caught up, resulting in comparable efficacy by day 21.

The observed overlap in efficacy between TCBZ and TETRA by day 21 suggests that initial differences in reduction rates were not maintained, possibly due to differences in the pharmacokinetic and pharmacodynamic properties of the drugs. TCBZ may have a faster onset, leading to a rapid reduction in parasite load, while TETRA might exhibit a slower, more sustained effect, resulting in comparable efficacy by the end of the study period. Additionally, parasite adaptation and delayed responses to TETRA could contribute to this convergence, as could metabolic differences affecting drug action over time. This overlap has clinical implications, as both drugs ultimately achieve similar outcomes, making other factors such as cost, side effects, and dosing convenience important considerations in treatment choice. Expanding on these aspects provides a well-rounded interpretation of why TCBZ and TETRA demonstrate overlapping efficacy at the study's conclusion.

#### 3.1.2 Comparative effects of treatment on eggs per gram count

The results presented in Tables 4, 5 provide a comparative analysis of treatment and time effects on eggs per gram (EPG) counts, showing distinct patterns across the treatment groups and time intervals. Specifically, Table 6 highlights significant differences between Albendazole (ALBE) and both Tetraclozan (TETRA) and Triclabendazole (TCBZ), with *p*-values below 0.05, indicating that ALBE was less effective in reducing EPG compared to TETRA and TCBZ. However, the difference between TETRA and TCBZ was not statistically significant (*p* > 0.05), suggesting comparable effectiveness between these two treatments by day 21. Table 7 further shows that time had a significant impact on EPG counts across all groups, with substantial reductions observed at each time interval (0, 7, 14, and 21 days). Notably, the EPG counts decreased progressively over time, with significant differences between each consecutive time point (*p* < 0.05). This time effect reinforces the efficacy of treatments over the study period, especially for TETRA and TCBZ, which showed similar outcomes by day 21, as noted in the overlap. Together, these tables emphasize that while ALBE was less effective overall, TETRA and TCBZ provided similar reductions in EPG by the study's end, and time consistently influenced EPG reductions across all groups.

**Table 6:** Comparative effects of treatment on eggs per gram count.

**Treatment _I_**	**Treatment _J_**	**Mean difference**	**SE**	***p*-Value**	**95% CI difference**	
					**Lower bound**	**Upper bound**
Albendazole	Tetraclozan	200.0	45.0	0.00	87.5	312.5
	Triclabendazole	230.5	45.9	0.00	116.0	344.9
Tetraclozan	Albendazole	−200.0	45.0	0.00	−312.5	−87.5
	Triclabendazole	30.5	45.9	1.00	−84.0	144.9
Triclabendazole	Albendazole	−230.5	45.9	0.00	−344.9	−116.0
	Tetraclozan	−30.5	45.9	1.00	−144.9	84.0

**Table 7:** Comparative effects of time on eggs per gram count.

**Time _I_**	**Time _J_**	**Mean difference**	**SE**	***p*-Value**	**95% CI difference**	
					**Lower bound**	**Upper bound**
0 day	7 day	1,561.746	61.033	0.000	1,392.540	1,730.952
	14 day	1,725.556	61.073	0.000	1,556.239	1,894.872
	21 day	1,779.048	59.970	0.000	1,612.791	1,945.304
7 day	0 day	−1,561.746	61.033	0.000	−1,730.952	−1,392.540
	14 day	163.810	18.775	0.000	111.759	215.860
	21 day	217.302	22.720	0.000	154.314	280.289
14 day	0 day	−1,725.556	61.073	0.000	−1,894.872	−1,556.239
	7 day	−163.810	18.775	0.000	−215.860	−111.759
	21 day	53.492	11.214	0.000	22.403	84.581
21 day	0 day	−1,779.048	59.970	0.000	−1,945.304	−1,612.791
	7 day	−217.302	22.720	0.000	−280.289	−154.314
	14 day	−53.492	11.214	.000	−84.581	−22.403

#### 3.1.3 Comparative effects of time on eggs per gram count

In all Groups, time had a significant effect on the EPG count. As shown in Table 5, there was a significant variation over time among the three treatment Groups (*p* < 0.05).

### 3.2 Biochemical analysis

In Group I, the levels of liver enzymes (AST, ALT, ALP, and GGT) before treatment were elevated above the normal range (Table 8). After treatment, the deviation of enzymes was recorded. However, the levels of these enzymes did not return to the normal range on day 7. The levels of ALT and GGT deviated on days 7 and 14 but were not within the reference range, whereas according to the reference range, the levels of AST and ALP became oriented on day 14. After 21 days, the level of enzymes returned to within the normal range. The values of TP and Alb before treatment deviated from the normal range. After treatment, the levels of TP and Alb did not return to within the normal range at 7 days, but at 14 and 21 days, both TP and Alb returned to within the normal range. Overall, while there was a gradual improvement in liver function indicators following treatment, full normalization for both liver enzymes and protein levels occurred by day 21.

**Table 8:** Mean ± standard deviation of biochemical parameters.

**Group**	**Date**	**AST (U/L)**	**ALT (U/L)**	**ALP (μmol/L)**	**GGT (U/L)**	**TP (g/dl)**	**Alb (g/dl)**
TCBZ	0	141.67 ± 6.64	68.13 ± 7.40	168.67 ± 5.30	65.13 ± 5.70	3.1 ± 0.32	1.99 ± 0.12
	7	131.27 ± 5.20	58.73 ± 7.55	159.47 ± 3.58	55.20 ± 5.82	5.40 ± 0.34	2.58 ± 0.27
	14	**106.20** ±8.42^*^	46.23 ± 4.60	**141.60** ±24.80^*^	44.53 ± 2.92	**6.27** ±0.27^*^	**3.23** ±0.21^*^
	21	**91.40** ±23.50^**^	**39.33** ±3.9^**^	**129.47** ±23.24^**^	**33.13** ±6.4^**^	**7.4** ±0.40^**^	**3.60** ±0.13^**^
TETRA	0	143.40 ± 7.30	58.80 ± 5.52	168.27 ± 7.50	74.53 ± 5.43	2.67 ± 0.39	2.00 ± 0.15
	7	136.47 ± 5.53	51.67 ± 4.35	160.13 ± 5.90	59.47 ± 3.31	4.87 ± 0.40	2.40 ± 0.11
	14	124.47 ± 8.75	**44.0** ±4.96^*^	**146.27** ±14.1^*^	**42.73** ±2.99^*^	**5.98** ±0.25^*^	**3.33** ±0.21^*^
	21	**101.33** ±20.70^**^	**38.6** ±5.15^**^	**137.87** ±21.2^**^	**31.47** ±3.91^**^	**7.13** ±0.4^**^	**3.70** ±0.10^**^
ALBE	0	150.67 ± 6.63	61.6 ± 33.18	172.00 ± 5.0	62.67 ± 10.50	3.20 ± 0.12	2.10 ± 0.10
	7	140.60 ± 5.82	53.80 ± 3.60	164.00 ± 3.8	53.07 ± 4.83	4.64 ± 0.16	2.30 ± 0.20
	14	130.07 ± 5.24	46.47 ± 2.33	156.67 ± 4.4	45.33 ± 3.60	5.70 ± 0.18	2.60 ± 0.13
	21	**117.40** ±26.76^*^	**42.73** ±2.5^*^	**151.60** ±6.9^*^	**39.93** ±5.40^*^	**6.00** ±0.24^*^	**2.80** ±0.24^*^
Reference range		**49_123**	**15_44**	**27_156**	**20_44**	**5.9_7.8**	**2.7_3.7**

All the bold values indicate samples with significant differences.

^**^More significant difference.

^*^Significant difference.

In Group II, as in Group I, all enzymes were elevated before treatment above the normal range. None of the enzyme levels returned to within the normal range on day 7; however, as Table 4 illustrates, after treatment on day 14, ALT, GGT and ALP were within the reference range, whereas AST was not. According to the reference range, all enzymes returned to the normal range on day 21. The values of TP and Alb deviated from the normal range before treatment (on day 0). After treatment, the levels of TP and Alb did not return to within the normal range at 7 days, but at 14 and 21 days, both TP and Alb returned to within the normal range.

**Figure 3:**
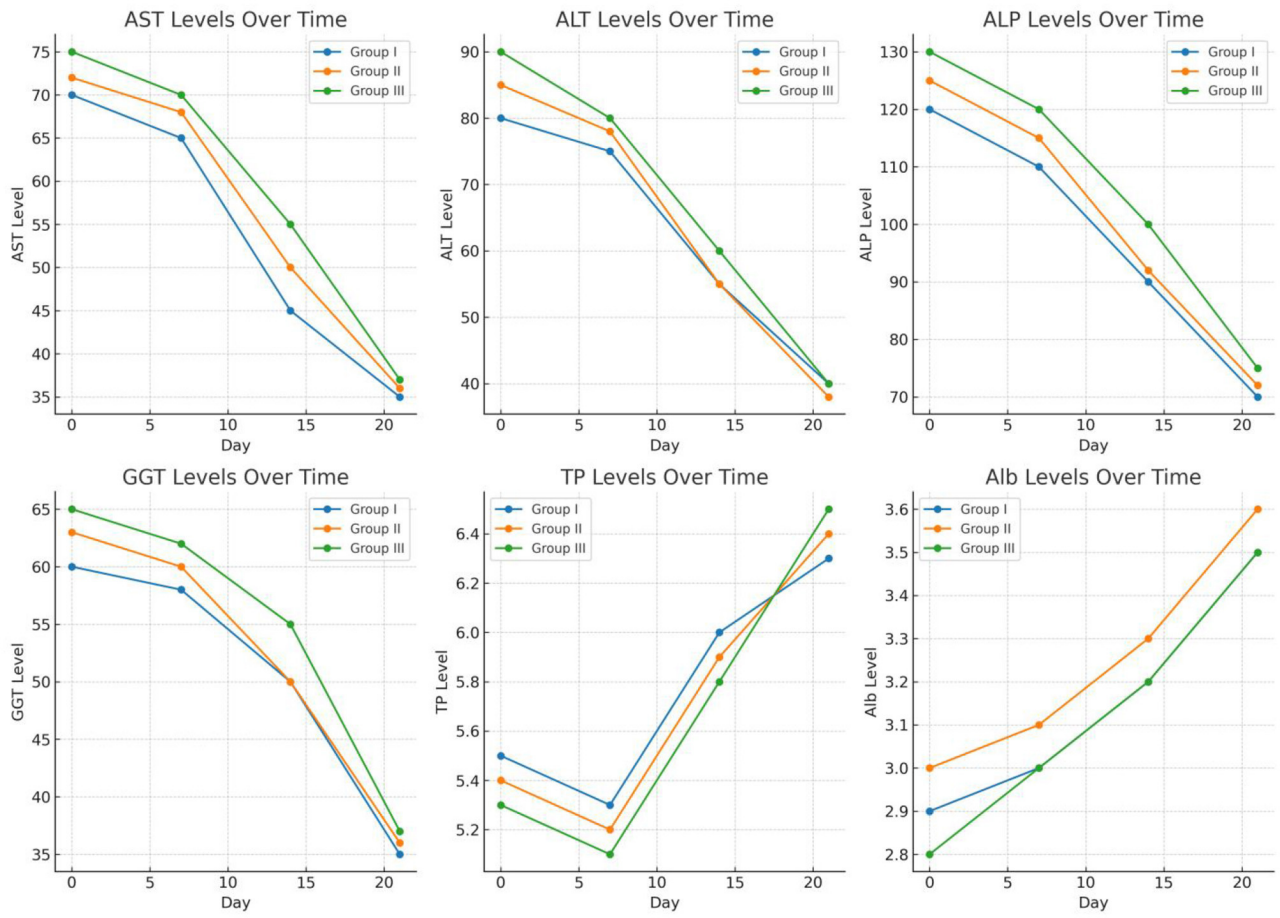
The biochemical parameter trends over time.

In Group III, the AST, ALT, ALP, and GGT levels before treatment were elevated above the normal values according to the reference ranges. After treatment, the levels of all enzymes, Tp and Alb, did not return to the normal range on days 7 and 14, whereas on day 21, all parameter values returned to the normal range. As shown in Table 4, in all the treatment Groups, the levels of the biochemical parameters gradually changed from 0 days to 21 days (Figure 3).

These lines graph displaying the biochemical parameter trends over time (days 0, 7, 14, and 21) for Groups I (TCDZ), II (TETRA), and III (ALBE). Each subplot represents one parameter AST, ALT, ALP, GGT, TP, and Alb showing how levels gradually approach normal ranges across the observation period. This visualization highlights the decreasing pattern of enzyme levels and the normalization of protein levels over time, providing a clear comparative view of recovery trends for each treatment group.

#### 3.2.1 Comparative effects of treatments with biochemical parameters

**Figure 4:**
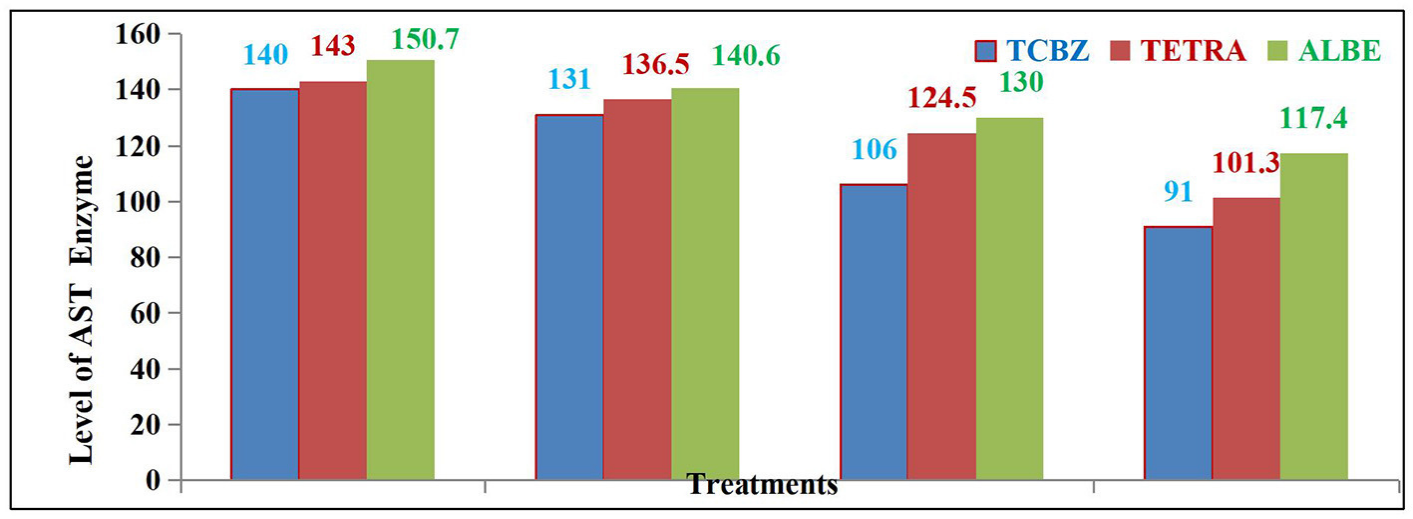
Comparison of AST activity among the three treatment Groups.

After 14 and 21 days of treatment, the average AST activity was significantly affected by the TCBZ (blue color) treatment. While ALBE (green color) had fewer effects than TETRA (pink color), after 21 days of treatment, the average value of the AST enzyme was within the normal range (as shown in Figure 4).

**Figure 5:**
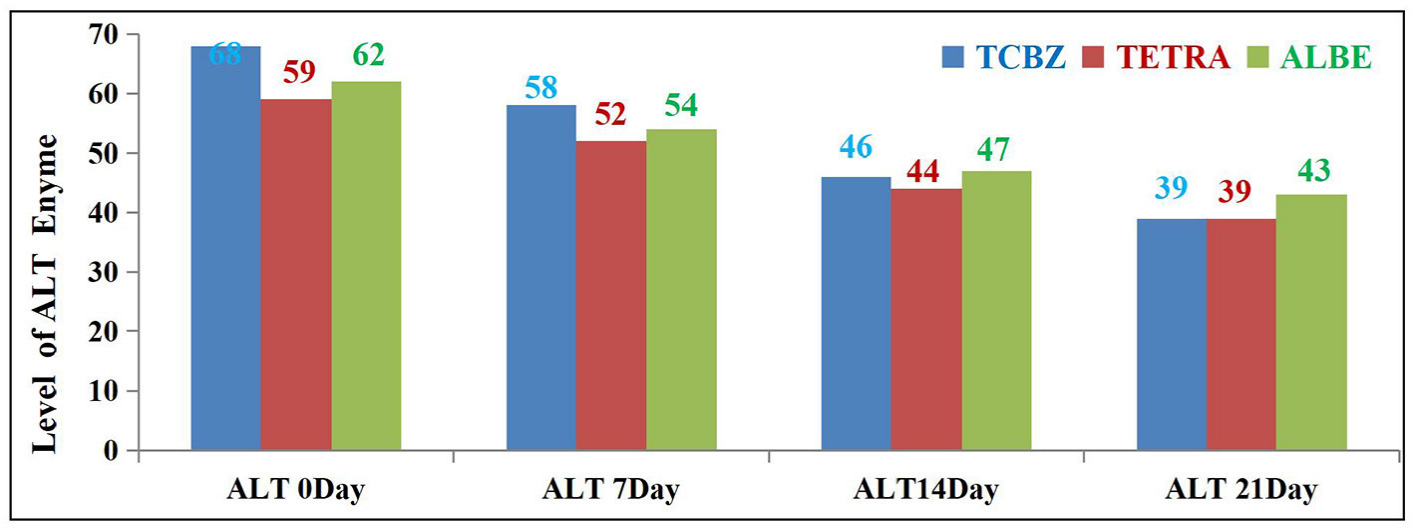
Comparison of ALT enzyme activity among the three treatment Groups.

On days 14 and 21 of treatment, both TCBZ (blue) and TETRA (pink) had visible effects on the average value of the ALT enzyme; however, the effects of TCBZ and TETRA were stronger than those of ALBE (green), but on day 21, the average ALT enzyme value was reduced to the reference range (as shown in Figure 5).

**Figure 6:**
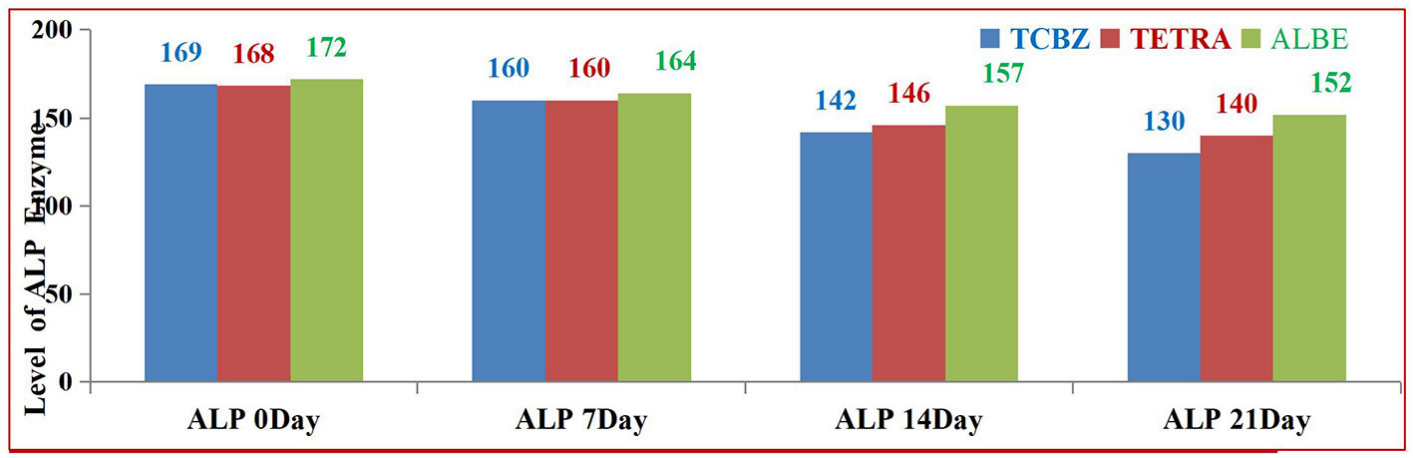
Comparison of ALP enzyme activity among the three treatment Groups.

According to Figure 5, TETRA exhibited greater effects than ALBE across the treatment Groups; however, on day 21, the average value of the ALP enzyme was corrected and remained within the normal range. On days 14 and 21 of treatment, TCBZ had the greatest effect on the average value of the ALP enzyme (Figure 6).

**Figure 7:**
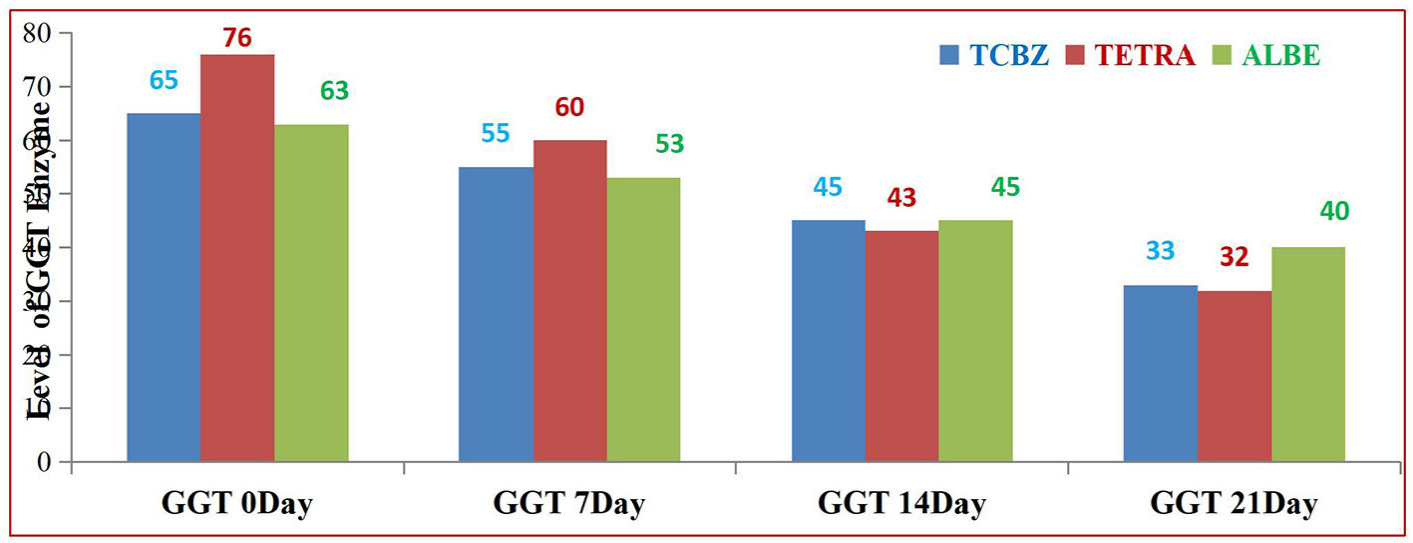
Comparison of GGT enzyme activity among the three treatment Groups.

Among the treatments, TETRA had the greatest effect on the average value of the GGT enzyme at 14 and 21 days of treatment. TCBZ also had greater effects than ALBE did; however, at 21 days of treatment, the average value of the GGT enzyme was within the normal range (Figure 7).

**Figure 8:**
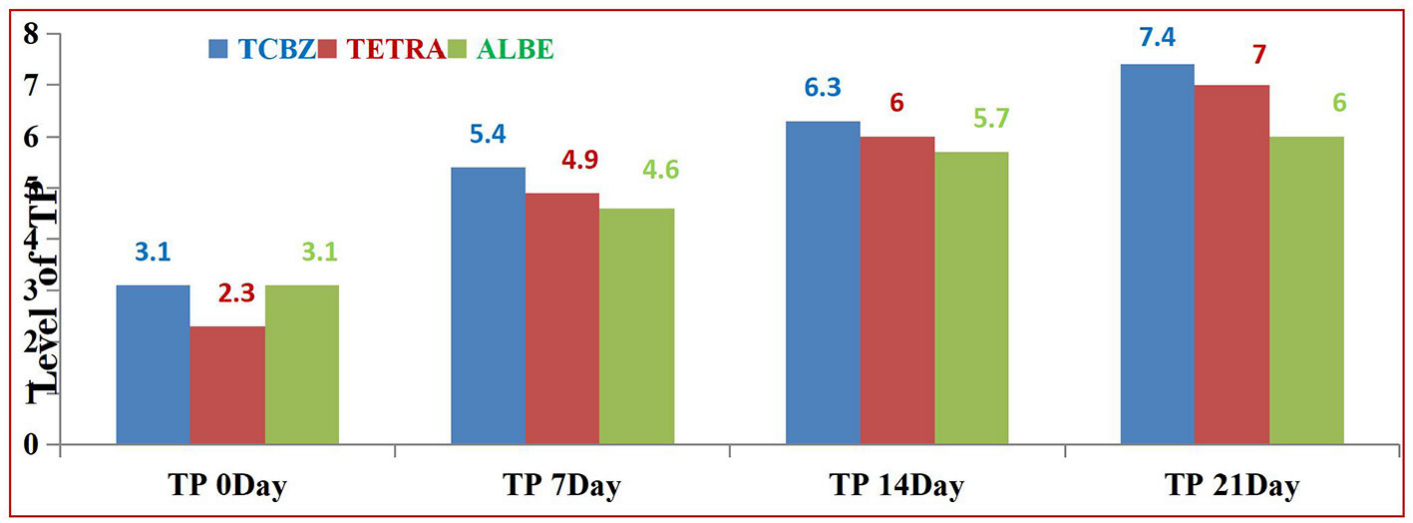
Comparison of total protein content among the three treatment Groups.

Among the treatment Groups, TCBZ had the greatest effect on the average value of TP at 14 and 21 days of treatment. TETA also had greater effects than ALBE did; however, at 21 days of treatment, the average value of TP was within the normal range. In all the treatment Groups, the average values of TP increased with respect to time (indicated in Figure 8).

**Figure 9:**
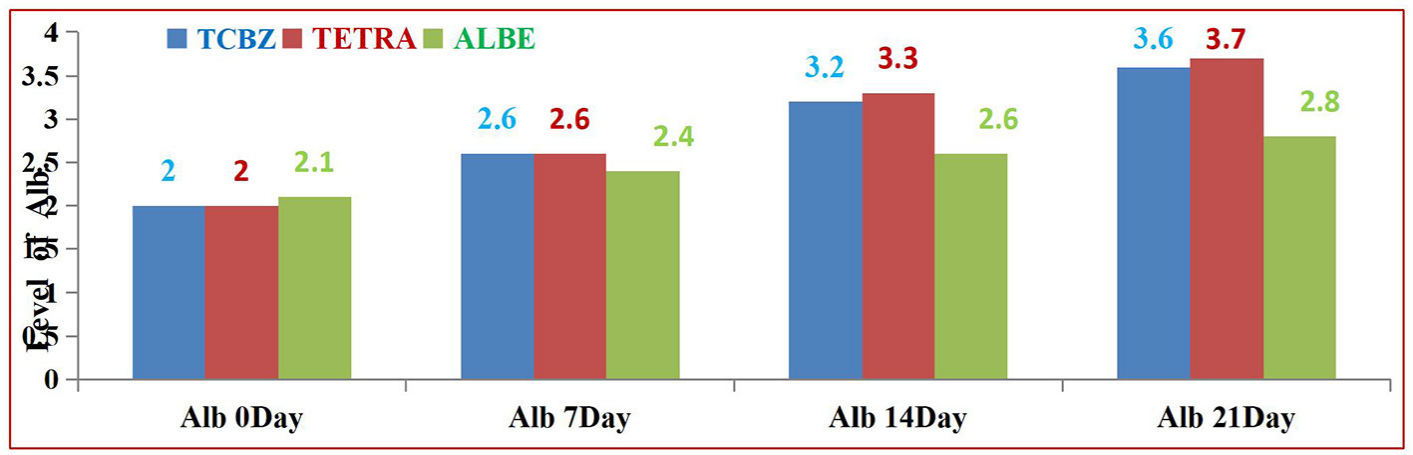
Comparison of the serum ALB concentration among the three treatment Groups.

Among the treatment Groups, TETRA had the greatest effect on the average value of Alb at 14 and 21 days of treatment. TCBZ also had a greater effect than ALBE did; however, at 21 days of treatment, the average value of Alb was within the normal range. In all treatment Groups, the average values of Alb increased with respect to time (indicated in Figure 9).

### 3.3 Adverse effects and treatment safety

While the primary objective of the trial was to evaluate the efficacy of Triclabendazole (TCBZ), Tetraclozan (TETRA), and Albendazole (ALBE) in treating *F. hepatica* infections, it is also important to consider the safety profile of these treatments. During the trial, the sheep were closely monitored for any potential adverse effects or side effects resulting from the administration of the anthelmintic drugs. Clinical observations were recorded at all time points (pre-treatment, 7, 14, and 21 days post-treatment), and no significant acute adverse reactions (such as vomiting, severe lethargy, or anaphylactic responses) were observed immediately following the treatment administration. However, mild side effects were noted in a small proportion of the sheep across the different treatment groups. These included transient gastrointestinal disturbances such as diarrhea and mild colic, which were not considered severe and generally resolved within a few days post-treatment. These effects were expected, as gastrointestinal upset is a known, but relatively mild, side effect of many anthelmintic drugs. In addition, the sheep in the Albendazole (ALBE) group showed a slight decrease in appetite in the initial 3–4 days after treatment, which was transient and did not lead to long-term weight loss or clinical complications.

It is important to note that no significant liver toxicity or abnormal changes in biochemical parameters (such as AST, ALT, ALP, and GGT) were observed in any of the treatment groups, suggesting that the anthelmintic drugs used in the study did not cause notable liver damage. However, the study did not specifically monitor for more subtle chronic effects that may take longer to manifest, and further long-term studies may be needed to fully evaluate the safety profile of these drugs in sheep under field conditions.

### 3.4 Clinical presentations before and after treatment

The clinical signs of fasciolosis, including diarrhea, pale mucous membranes, bottle jaw, and depression, were observed before and after treatment, and the percentage improvement was calculated for each symptom across the three treatment groups: Triclabendazole (TCBZ), Tetraclozan (TETRA), and Albendazole (ALBE). The results are summarized in Table 9 below, which shows the pre-treatment prevalence of each symptom and the post-treatment improvement (calculated as the percentage of animals that improved by day 21).

**Table 9:** Percentage improvement in clinical symptoms by treatment group.

**Clinical signs**	**Pre-treatment prevalence (%)**	**Group I (TCBZ) improvement (%)**	**Group II (TETRA) improvement (%)**	**Group III (ALBE) improvement (%)**
Diarrhea	60 (Group I)	93.3%	87.5%	90.9%
	53 (Group II)			
	73 (Group III)			
Pale mucous membranes	100 (all groups)	100%	100%	100%
Depression	80 (Group I)	92.3%	93.3%	91.7%
	80 (Group II)			
	73 (Group III)			

This table illustrates that all three treatments led to substantial clinical improvement in the sheep. In particular, symptoms such as diarrhea and depression showed high recovery rates across the groups, with improvements of over 90% in all treatment groups. The symptom of bottle jaw also showed considerable improvement, especially in Tetraclozan (TETRA) and Triclabendazole (TCBZ) groups, where the improvement was around 80%−85%. Pale mucous membranes, a consistent clinical sign, showed full recovery (100% improvement) in all groups, which suggests a rapid response to the treatments in alleviating severe anemia associated with fasciolosis.

### 3.5 Variability in clinical and biochemical responses

The overall clinical and biochemical improvements were significant across all treatment groups, but responses were not uniform across all animals. A variety of clinical and biochemical outcomes was noted within each treatment group, reflecting natural biological variation and the potential influence of individual factors such as weight, age, or infection severity. For clinical improvement, the percentage of sheep showing improvement in each symptom (such as diarrhea, pale mucous membranes, bottle jaw, and depression) was calculated based on the number of animals in each group that showed visible recovery by day 21. While the majority of animals in each group showed substantial improvement (with recovery rates ranging from 80% to 100%), there was some variability in the response rates. For example, in Group II (TETRA), while most sheep showed significant recovery from bottle jaw (85.7% improvement), a few individuals (around 2–3 sheep) exhibited minimal change, which contributed to the overall group variability. Similarly, diarrhea and depression showed higher overall recovery rates, but a few animals in each group exhibited persistent symptoms that did not improve within the study period.

In terms of biochemical data, variability was also observed in the liver biomarkers (such as AST, ALT, ALP, and GGT) measured throughout the trial. For instance, while the majority of animals in all treatment groups exhibited a significant decrease in liver enzyme levels, a subset of animals (~2–4 sheep per group) showed persistently elevated liver enzyme levels, which might indicate a delayed effective response to treatment. Such variability is not unusual in field trials involving naturally infected animals, where factors such as the degree of parasitic load, preexisting liver conditions, or individual immune responses can influence treatment outcomes.

## 4 Discussion

Economic losses from liver condemnation and animal suffering caused by parasitic infections pose significant challenges to livestock health and meat production globally. Between 2018 and 2021, Ali et al. (16) conducted a survey at the Central Muscat Municipality Slaughterhouse, Sultanate of Oman, to assess parasitic infections in 948 condemned sheep livers. Pathological and parasitological examinations identified *Dicrocoelium dendriticum, Cysticercus tenuicollis, Stilesia hepatica, Fasciola hepatica*, and hydatid cysts. Fasciolosis remains one of the most important helminthic diseases of livestock in many countries worldwide. Fascioliasis, a globally distributed zoonotic disease caused by *Fasciola hepatica* and *Fasciola gigantica*, affects livestock and humans, primarily through ingestion of contaminated food or water [Gandhi et al. (17)].

In Ethiopia, it is also one of the major constraint factors for ovine production development because it infers direct and indirect losses in different parts of the country. Ovine fasciolosis infection is chronic and has a major economic impact due to reductions in weight gain, lamb birth, milk yield and fertility and liver damage (18). This study aimed to compare the effectiveness of Tetraclozan, Albendazole, and Triclabendazole on ovine fasciolosis and its effects on biochemical parameters. Although triclabendazole (TCBZ) resistance is a heritable trait, primarily governed by a single locus with dominant inheritance (19), it remains effective against liver fluke in this study. The results clearly revealed that Triclabendazole, Tetraclozan and Albendazole were highly effective, effective and poorly effective compounds for the treatment of *Fasciola* spp. in this field trial, respectively. In the present study, Triclabendazole had significant efficacy (97.8%) against *Fasciola* species, which is not in agreement with previous studies from Australia (20), Netherlands (21) and Scotland (22), indicating that *Fasciola* is less effective than Triclabendazole (TCBZ) in sheep because of the extensive and prolonged use of Triclabendazole (23). According to the present findings, the efficacy of Albendazole (84%) indicates resistance or treatment failure, as efficacy below 90% is considered inadequate based on the standards set by the World Association for the Advancement of Veterinary Parasitologists (WAAVP) (11).

In contrast, the current study found a higher efficacy for triclabendazole (TBZ) at 97.8%, which slightly exceeds the 95.6% efficacy reported for TBZ alone on post-treatment day 28. Additionally, a combination of triclabendazole and levamisole achieved a fecal egg count reduction (FECR) of 97.33% on day 28, consistent with the findings of Tabari et al. (24). Our finding contrasts with the report by Flanagan et al. (25), which documented a lower efficacy of triclabendazole (TBZ) at 72.2% against liver flukes. A lack of uniformity in results could be explained by differences in study design, parasite strains, and environmental conditions. There may also be differences in experimental protocols, such as treatment regimens and sample sizes, as well as genetic differences between Fasciola species across regions. Moreover, environmental factors, including climate, farming practices, and livestock management, likely influence the effectiveness of treatments. These factors highlight the challenges in making direct comparisons between studies, underscoring the need for more standardized approaches in future research to improve the consistency and applicability of findings across different settings.

The present findings are in line with those of field studies on the use of Triclabendazole against mixed age fluke infection in sheep (26) and a study conducted at Mekelle University by (27), who reported that Triclabendazole has 100% efficacy against *Fasciola* infection, and a previous study performed at Bonga Sheep Breeding and Improvement Center, Southwest Ethiopia, by (18), who reported that Triclabendazole has 96.51% and 97.18% efficacy against *Fasciola* infection after treatment at 14 and 21 days, respectively. Studies have reported varying results: Babják et al. (15) found a 77%−81.8% egg reduction after 14 days of treatment, while Marcos et al. (28) reported a 95% cure rate in humans using 10 mg/kg of triclabendazole (TBZ). Similarly, efficacy ranged from 29% for Albendazole to 100% for TBZ (29).

This might be because Triclabendazole works against both adult and juvenile flukes. Its potency against immature flukes is noteworthy since the fluke is at its most harmful stage of infection during this time, when it migrates and invasive into tissue (30). Differences in treatment efficacy may stem from regional variations in Fasciola strains, which exhibit diverse susceptibility to drugs. Genetic diversity, environmental factors (e.g., climate, farming practices), and livestock management can all influence the parasite's lifecycle and drug effectiveness. Additionally, treatment timing, dosage, and co-infections may further impact outcomes. Addressing these factors could clarify efficacy discrepancies. Moreover, the development of resistance in Fasciola spp. may also contribute to the observed discrepancies, highlighting the need for ongoing surveillance and alternative treatment strategies to manage this parasitic infection effectively.

In the present study, Albendazole had a limited effect on fasciolosis, as its administration decreased the EPG count, but the eggs did not disappear on the last days of treatment. The low effectiveness of Albendazole in this study (84%) is in line with the findings of Albendazole resistance reported in the Korem and Hashengae areas of Ethiopia (27), Spain, and Egypt (31–33). The present findings are also consistent with those of Kouadio et al. (34), who reported that triclabendazole is highly effective treatment for liver fluke infections in cattle in a region of Côte d'Ivoire with a high prevalence of fascioliasis compare with albendazole. Even if the percentage reduction remains below 90%, Albendazole can still decrease the fluke burden to a level that is advantageous for both animal health and economic viability (35).

The reason why eggs were still released in this treatment Group could be that Albendazole affects only the adult stages of the *Fasciola* species, whereas the immature stages evolve into adult stages. Despite receiving Albendazole medication, the majority of treated patients passed eggs in their feces following treatment; this could be explained by the low effectiveness of the drug. Another factor could be the frequent and unsystematic use of Albendazole by farmers in the study area, which may have contributed to reduced effectiveness over time due to resistance development. Other factors that may lead to an underestimation of the actual value include variations in egg distribution within a single fecal sample, daily changes in the host's fecal production and consistency, and fluctuations in the parasite's oviposition patterns (36).

In the present study, Tetraclozan (oxyclozanide combined with tetramizole) clearly showed high effectiveness against fasciolosis, resulting in a 97.8% reduction in FECR on day 14 post treatment and up to a 99% reduction in FECR on day 21, which is in agreement with the results of a previous study on the use of Tetraclozan against *Fasciola* spp. in Tanzania (37) and Pakistan (38), which used Tetraclozan (oxyclozanide combined with tetramizole) as a flukicide with an efficiency of 96%. Tetraclozan might have high efficacy because it inhibits ATP generation and blocks oxidative phosphorylation in parasite mitochondria, ultimately leading to parasite death from abnormalities related to energy metabolism (39–42).

This disruption in energy metabolism leads to significant cellular dysfunction and ultimately results in the death of both adult and immature stages of *Fasciola* species. By targeting multiple developmental stages of the parasite, combined tetramisole with oxyclozanid ensures a more comprehensive approach to treatment, reducing the chance of reinfection from immature stages that may survive other treatments. In contrast, albendazole primarily targets the adult stages of the parasite by interfering with tubulin polymerization, which is crucial for maintaining the structure and function of the parasite's cytoskeleton.

The results of the present study also revealed that the elevation of liver enzymes and the decreases in Tp and Alb prior to treatment are in line with those reported in previous investigations, in which hepatocellular injury was linked to increases in the levels of aspartate aminotransferase (AST) and alanine aminotransferase (ALT), and potential damage to the hepatobiliary system was the cause of increases in alkaline phosphatase (ALP) and gamma glutamyl transferase (GGT) levels (43). The current results corroborate those of a prior study in which infected sheep had high levels of the blood enzymes AST and ALT, potentially as a result of significant liver parenchymal damage, hepatotoxic production, and cellular necrosis. Moreover, cholestasis and bile duct injury may be linked to significant increases in GGT and ALP levels. GGT is released into the bloodstream when the bile duct epithelium is damaged, which increases the serum level of GGT, primarily after flukes have penetrated the bile duct (44).

The present findings were in agreement with the results of earlier investigations, and the mean values of all biochemical parameters that were evaluated in this study were found to be above the reference ranges. Early infection-related elevations in serum transaminase (ALT and AST) levels may be associated with hepatic necrosis and degenerative alterations caused by juvenile flukes migrating through the liver parenchyma (45), whereas (46) reported considerably reduced AST, ALT, and ALP activity in sheep serum from infected Groups, which contradicts our findings.

In this study, the total protein and albumin levels of sheep with *F. hepatica* were found to be low, which is in line with the results of previous investigations (46, 47). This could be the result of liver damage because the liver is crucial for the formation of both proteins and albumin. The damage that juvenile flukes cause to liver cells is indicated by the levels of albumin and total serum proteins (48). Gradual decreases in the mean values of AST, ALT, GGT, and ALP were recorded after treatment, along with gradual increases in the mean values of TP and Alb in all treatment Groups; this might be due to the effects of treatment on biochemical parameters. These results are consistent with those of previous studies (49). The delayed normalization of liver related biochemical parameters, such as AST, ALT, GGT, and ALP, suggests a gradual hepatic recovery post treatment, with full return to baseline only by day 21. This may reflect the severity of initial liver damage or the prolonged hepatic impact of juvenile flukes. Monitoring liver enzyme levels over an extended period post-treatment is essential for assessing full hepatic recovery and treatment efficacy. Elevated enzyme levels in this study contrast with previous findings, likely due to differences in infection duration, parasite load, *F. hepatica* strain, or the general health of animals. Considering these variables can improve understanding of hepatic enzyme responses across studies.

The present study demonstrates that treating infected animals with Triclabendazole (TCBZ), Tetraclozan (TETRA), and Albendazole (ALBE) effectively restored altered biochemical parameters to within normal reference ranges, likely due to the flukicidal action of these drugs. Liver fluke infections, such as fasciolosis, are known to disrupt liver function, leading to changes in biochemical markers, including elevated levels of liver enzymes (e.g., AST, ALT), bilirubin, and total protein, which serve as indicators of liver damage and metabolic imbalance (50). Effective treatment not only eliminates the parasites but also allows the liver to regenerate and resume normal function, leading to a normalization of these biochemical parameters (46). By 21 days post-treatment, all measured biochemical parameters in the current study had returned to normal ranges, suggesting that the treatments alleviated the physiological stress and liver damage caused by the fluke burden. The efficacy of TCBZ, TETRA, and ALBE in reducing the parasitic load likely contributed to this recovery, as effective anthelmintics minimize liver damage by preventing further tissue destruction and inflammation (15, 24). This observation is consistent with previous studies that reported significant improvements in biochemical profiles following successful treatment. For instance, TCBZ has been widely recognized as highly effective against both adult and immature flukes, allowing rapid recovery of liver function post-treatment (51). Additionally, studies by Sanabria and Romero (29) highlighted the broad-spectrum efficacy of ALBE and TCBZ, further supporting their role in restoring metabolic homeostasis in infected animals. These findings emphasize the importance of timely and effective treatment of fasciolosis to mitigate systemic impacts and restore the health and productivity of affected animals. Monitoring biochemical parameters during and after treatment provides a valuable tool for assessing treatment efficacy and the overall recovery process. In all the Groups, treatment and time had a significant effect on the concentrations of the biochemical parameters (*p* < 0.05), possibly because of the effects of the treatment (46).

The current findings also revealed that the fecal egg count reduction rates and the tested biochemical parameters were related. As the FECR increased, the biochemical parameters returned to within the normal range. In Groups-I and-II, the mean values of the biochemical parameters were corrected as the egg reduction rate increased, whereas in Group III, the mean values of the biochemical parameters were not strongly corrected on days 7 and 14, when the egg reduction rates were below the expected percentage. The intervention administered to Group III may have been less effective in reducing the parasite load, as indicated by the lower egg reduction rates. This reduced efficacy could lead to insufficient improvement in biochemical parameters, which often reflect ongoing parasitic infections or liver impairment. Some treatments may require more time to produce significant changes in biochemical markers. As a result, the biochemical indicators may not have fully captured the treatment effects by days 7 and 14, necessitating additional time to show notable improvements. Persistent high parasite load might cause prolonged liver dysfunction or other biochemical imbalances. Additionally, variability in individual responses within Group III could affect the overall mean values of biochemical parameters, with some animals showing a slower or less pronounced response, thereby impacting the group's average results.

In general, In the present study, Albendazole had a limited effect on fasciolosis, with an efficacy of 84%, which is consistent with reports of reduced efficacy in regions with a long-term history of Albendazole use for fasciolosis control. This aligns with findings from studies on cattle farms with a prolonged history of Albendazole use, where resistance was observed. For instance, a study conducted in 2020 on Charolais cows reported a fecal egg count reduction of 77%−81.8% on day 14 post-treatment, highlighting a suboptimal response to Albendazole (15). Our study showed triclabendazole (97.8%) as the most effective treatment, but long-term control relies on vaccination, with experimental vaccines demonstrating 32%−100% efficacy. Biological snail control, like using *Sphaerodema urinator*, could enhance chemical and immunological strategies for integrated fasciolosis management (52).

However, despite the promising efficacy of TCBZ, its widespread use is threatened by the emergence of resistance. Triclabendazole resistance has been reported globally, affecting treatment outcomes and complicating control efforts. Recent studies, such as Beesley et al. (19), have identified specific genomic loci and candidate genes associated with TCBZ resistance, including those involved in drug transport (ABCB1), signal transduction, and drug sequestration (FABP). These insights into the genetic mechanisms of resistance underscore the need for molecular surveillance to monitor resistance and inform the development of alternative treatments and sustainable control strategies. Moreover, the effectiveness of triclabendazole has been further confirmed by a recent clinical trial conducted in Côte d'Ivoire, which demonstrated significantly higher efficacy in cattle treated with triclabendazole (95.4% non-egg shedding rate) compared to albendazole (70.3%) (34). These findings reinforce the critical role of TCBZ in livestock fascioliasis management. However, its availability remains limited to certain regions, underscoring the need for broader access to the drug. In regions where TCBZ is available, it should be combined with strategies such as snail control and farmer education to prevent reinfection and ensure more sustainable control of the disease. Also A recent study comparing triclabendazole (TBZ) and TBZ combined with levamisole (LVM) in sheep naturally infected with *Fasciola* spp. found that both treatments significantly reduced fecal egg counts (FEC), with TBZ + LVM showing superior results at 7 and 14 days post-treatment (*p* < 0.05) by Tabari et al. (24). The combination treatment also achieved a greater fecal egg count reduction (FECR) by day 28, exceeding 90%, highlighting its potential to improve treatment efficacy and slow resistance development. This indicated that a combination treatment resulted in greater fecal egg count reduction (FECR) by day 28, reaching values higher than 90%, supporting the potential of this strategy to enhance treatment efficacy and slow down resistance development.

The present study also revealed that the observed clinical presentations improved after treatment. Among the observed clinical signs, diarrhea, depression, and bottle jaws at 7, 14, and 21 days post-treatment improved as the animals recovered, whereas the pale mucous membrane did not improve immediately after treatment. The percentage improvement in the clinical signs indicated that in Group I (TCBZ), most of the animals were clinically improved compared with those in Group II (TETRA) and Group III (ALBE), and a good percentage improvement in the clinical presentation was observed in Group II compared with that in Group III, which might be due to the effectiveness of the treatment accordingly and the enhancement of liver metabolism, regeneration of hepatocytes, or lowering of the parasite burden (40). This difference in the rate of symptom improvement highlights an interesting aspect of treatment efficacy. The faster improvement in signs like diarrhea and depression may be due to the treatments' effects on reducing the parasite load and thereby alleviating immediate gastrointestinal and systemic symptoms. In contrast, the delayed improvement in pale mucous membranes suggests that anemia or reduced hemoglobin levels, often linked to chronic liver damage or sustained blood loss, may require a longer recovery period. This could indicate that while the treatments were effective in reducing parasitic effects, full hepatic and hematological recovery may take additional time, underscoring the importance of extended monitoring to capture the full scope of treatment efficacy.

Understanding the effectiveness of anthelmintics and their impact on treated animals is crucial for identifying optimal therapies, preventing drug resistance, and minimizing economic losses. The findings from this study will help veterinary professionals, clinicians, and drug dispensers select the most effective drugs. Additionally, policymakers, program developers, and researchers can use the results to improve public awareness and refine existing policies and strategies. Given the widespread occurrence of fasciolosis in the Dembiya district, effective control and treatment using selected anthelmintics are essential to prevent economic losses. The findings of Dag et al. (53) provide evidence supporting plant-based anthelmintics as viable alternatives, particularly for managing *Haemonchus contortus* infections. Similarly, the observed effectiveness of *Artemisia annua* aligns with our study's emphasis on identifying and validating alternative treatments to address resistance challenges associated with conventional anthelmintics like albendazole. Both *Haemonchus contortus* and *Fasciola* spp. require integrated management approaches to mitigate resistance. For *H. contortus*, this includes exploring alternative control methods alongside chemical treatments, which parallels the need for strategies like snail control and farmer education in fasciolosis management (54).

For future research, a more focused investigation into the mechanisms linking FECR and biochemical markers could provide insights into their potential to predict long-term treatment outcomes. Additionally, studies on drug resistance in parasites and the exploration of alternative therapeutic agents could improve treatment protocols, especially when biochemical recovery is delayed. Long-term monitoring of liver function parameters post-treatment would help assess whether initial improvements are sustained or if any residual liver dysfunction remains. Expanding the scope to include a wider range of anthelmintic drugs from diverse sources would allow for a more comprehensive evaluation of treatment options and reveal how manufacturing differences might affect therapeutic success.

In addition to evaluating the effectiveness of Triclabendazole, Tetraclozan, and Albendazole for ovine fasciolosis, addressing practical challenges in implementing these treatments on a larger scale is essential. Logistical issues, including the availability, proper storage, and distribution of anthelmintics particularly in remote areas must be addressed to ensure reliable access to effective treatments. Farmer education is also critical, emphasizing proper drug administration, preventing drug resistance, and adopting integrated parasite management strategies. Successful control of ovine fasciolosis requires not only accessible anthelmintics but also supportive infrastructure, policies, and community engagement to ensure long-term effectiveness and mitigate the economic impact of the disease.

### 4.1 Limitations and strengths of the study

The study had certain limitations that made it difficult to draw comprehensive scientific conclusions in the veterinary field across the entire pharmaceutical market region. One limitation of this study was that it did not consider the resistance of anthelmintics data. The exclusion of resistance data, for example, could mean that the observed efficacy of the drugs may not fully represent their performance in the presence of resistant parasite populations, which could lead to overestimation of treatment success. In a real-world setting where resistance is a significant concern, the drugs' efficacy may be lower than indicated by the study. Additionally, the study's conclusions regarding the effectiveness of the selected veterinary anthelminthic drug were influenced by the fact that it only compared limited brands from single manufacturing countries that exercise different manufacturing practices. The study excelled in addressing the identified gap and thoroughly answering the research questions. Moreover, this study is the first to investigate the effects of treatment on biochemical parameters such as liver enzyme profiles, total protein, and albumin in a veterinary setting, especially in terms of the efficacy of Triclabendazole, Tetraclozan, and Albendazole anthelmintic drugs. Despite facing challenges during the field trial study, the researcher demonstrated unwavering determination in tackling the study's objectives, even if there was peace insecurity (in life-threatening conditions). The present study's focus on a limited range of drugs triclabendazole, tetraclozan, and albendazole from specific manufacturing countries presents a potential limitation in assessing treatment efficacy. By concentrating on these particular agents, the study may not fully capture the broader spectrum of available anthelmintic treatments or consider the potential influence of variations in drug formulation, production standards, and active ingredient concentrations across different manufacturers. Such variations could significantly impact drug efficacy and bio-availability, potentially affecting treatment outcomes.

## 5 Conclusion and future perspective

Among the three anthelmintics tested against ovine fasciolosis, TCBZ proved to be the most effective, followed by TETRA, while ALBE demonstrated the least effectiveness. Significant reductions in *Fasciola* egg counts were observed across all treatment groups on days 0, 7, 14, and 21, with FECRT values of 97.8% for TCBZ, 96.6% for TETRA, and 84% for ALBE, respectively. Notably, TETRA treatment led to a marked decrease in *Fasciola* egg shedding after 14 days, with concurrent improvements in biochemical markers such as AST, ALT, ALP, and GGT, and increases in total protein and albumin levels over the 21-day period. These biochemical parameters proved to be reliable indicators for assessing the severity of ovine fasciolosis and evaluating the success of treatment, making them valuable for early diagnosis and ongoing monitoring of liver function. In terms of clinical improvements, the study observed significant recovery in clinical signs such as diarrhea, depression, and bottle jaws across all treatment groups, with the most pronounced improvements seen in the TCBZ-treated group, followed by TETRA, while the ALBE-treated group showed slower recovery. These findings highlight the importance of both biochemical and clinical markers in evaluating the effectiveness of treatment. Based on these results, TETRA presents a viable alternative when TCBZ is unavailable, offering a highly effective treatment option. Moreover, the use of biochemical markers for assessing liver damage and disease severity is recommended for comprehensive management of ovine fasciolosis. However, the study also underscores the need for continued research on anthelmintic resistance, particularly in different *Fasciola* species across Ethiopia, to inform future treatment strategies and prevent the development of resistance. Ultimately, these findings contribute to optimizing treatment protocols and enhancing the control of fasciolosis in ovine populations, with potential implications for improving livestock productivity and supporting sustainable farming practices.

## Data Availability

The raw data supporting the conclusions of this article will be made available by the authors, without undue reservation.
