# Supplementary material for: Comparative analysis of anthelmintic treatments: impact on liver biomarkers and clinical recovery in sheep with fasciolosis
Source: Front Vet Sci. 2025 Feb 20;12:1485568. doi: 10.3389/fvets.2025.1485568 (PMC11884322; doi:10.3389/fvets.2025.1485568)
Supplement: Supplementary file 1 [file Data_Sheet_1.pdf]

Supportive information EPG

Group 1 Individual egg counts on Mac master slides treated with TCBZ

| ANIMAL ID | 0 Day | 7 Day | 14 Day | 21 Day | FECRT with in Group 1 | | |
|---|---|---|---|---|---|---|---|
| | | | | | FECR 1 | FECR 2 | FECR 3 |
| TCBZ -1 | 2000 | 100 | 0 | 0 | 95.5% | 100% | 100% |
| TCBZ -2 | 2200 | 100 | 0 | 0 | 95% | 100% | 100% |
| TCBZ -3 | 1500 | 200 | 0 | 0 | 86.7% | 100% | 100% |
| TCBZ -4 | 1200 | 0 | 0 | 0 | 100% | 100% | 100% |
| TCBZ-5 | 1400 | 0 | 0 | 0 | 100% | 100% | 100% |
| TCBZ-6 | 1600 | 0 | 0 | 0 | 100% | 100% | 100% |
| TCBZ -7 | 1100 | 100 | 100 | 100 | 91% | 91% | 91% |
| TCBZ -8 | 1300 | 200 | 100 | 0 | 84.6% | 92.3% | 100% |
| TCBZ -9 | 1700 | 100 | 0 | 100 | 94% | 100% | 94% |
| TCBZ -10 | 2200 | 100 | 0 | 0 | 95% | 100% | 100% |
| TCBZ -11 | 1400 | 100 | 0 | 0 | 93% | 100% | 100% |
| TCBZ -12 | 2500 | 200 | 100 | 0 | 92% | 96% | 100% |
| TCBZ -13 | 1900 | 0 | 0 | 0 | 100% | 100% | 100% |
| TCBZ -14 | 1600 | 0 | 0 | 0 | 100% | 100% | 100% |
| TCBZ- 15 | 2300 | 0 | 0 | 0 | 100% | 100% | 100% |
| Average of EPG and FECRT | 1726.67 | 80.00 | 20.00 | 13.33 | 95% | 99% | 99% |

:Group -2 individual egg counts on mac master slides treated with TETRA

| ANIMAL ID | 0 Day | 7 day | 14 day | 21 day | FECRT with in Group 2 | | |
|---|---|---|---|---|---|---|---|
| | | | | | FECRT 1 | FECRT2 | FECRT |
| TETRA -1 | 2300 | 200 | 100 | 0 | 91.3% | 95.7% | 100% |
| TETRA-2 | 1000 | 200 | 100 | 0 | 80% | 90% | 100% |
| TETRA-3 | 2100 | 100 | 0 | 0 | 95% | 100% | 100% |
| TETRA-4 | 1800 | 0 | 0 | 0 | 100% | 100% | 100% |
| TETRA-5 | 2100 | 200 | 0 | 0 | 90.5% | 100% | 100% |
| TETRA -6 | 1500 | 100 | 0 | 0 | 93.3% | 100% | 100% |
| TETRA -7 | 2200 | 200 | 100 | 100 | 91.% | 95% | 95% |
| TETRA -8 | 1700 | 200 | 100 | 100 | 88% | 94% | 94% |
| TETRA -9 | 1300 | 100 | 0 | 0 | 92.3% | 100% | 100% |
| TETRA -10 | 1900 | 200 | 0 | 0 | 89.5% | 100% | 100% |
| TETRA -11 | 2200 | 100 | 0 | 0 | 95% | 100% | 100% |
| TETRA -12 | 1400 | 200 | 100 | 0 | 85.7% | 93% | 100% |
| TETRA -13 | 1600 | 100 | 0 | 0 | 93.8% | 100% | 100% |
| TETRA -14 | 2400 | 100 | 0 | 0 | 95.8% | 100% | 100% |
| TETRA -15 | 1600 | 100 | 0 | 0 | 93.8% | 100% | 100% |
| *Average of EPG and FECRT* | *1806.67* | *140.00* | *33.33* | *13.33* | *91.67%* | *97.85%* | *99%* |

*TETRA=Tetraclozan

Group -3. Individual egg counts on Mac master slides treated with ALBE

| Animal ID | 0 day | 7 day | 14 day | 21 day | FECRT with in Group 3 | | |
|---|---|---|---|---|---|---|---|
| | | | | | FECRT1 | FECRT2 | FECRT3 |
| ALBE- 1 | 2200 | 1000 | 200 | 100 | 54.5% | 91% | 95% |
| ALBE- 2 | 1600 | 800 | 300 | 200 | 50% | 81% | 87.5% |
| ALBE- 3 | 1800 | 600 | 400 | 200 | 66.7% | 77.8% | 88.9% |
| ALBE- 4 | 1500 | 400 | 300 | 200 | 73% | 80% | 86.7% |
| ALBE- 5 | 2200 | 800 | 400 | 200 | 63.6% | 81.8% | 91% |
| ALBE- 6 | 2400 | 1100 | 600 | 200 | 54% | 75% | 91.7% |
| ALBE- 7 | 1700 | 700 | 200 | 100 | 58.8% | 88.2% | 94% |
| ALBE- 8 | 2100 | 200 | 0 | 0 | 90.5% | 100% | 100% |
| ALBE- 9 | 2000 | 300 | 0 | 0 | 85% | 100% | 100% |
| ALB E- 10 | 1700 | 500 | 200 | 100 | 70.6% | 88% | 94% |
| ALBE- 11 | 2300 | 300 | 0 | 0 | 87% | 100% | 100% |
| ALBE- 12 | 1300 | 400 | 200 | 0 | 69% | 84.6% | 100% |
| ALBE- 13 | 2000 | 500 | 300 | 100 | 75% | 85% | 95% |
| ALBE- 14 | 1500 | 400 | 300 | 100 | 73.3% | 80% | 93.3% |
| ALBE- 15 | 2100 | 300 | 200 | 100 | 85.7% | 90.5% | 95% |
| Average of EPG and FECRT | *1893* | *553.3* | *240* | *106.6* | *70.4%* | *86.9%* | *94%* |

*ALBE=Albendazole

The average value s of biochemical parameters for the three groups

| The average value of biochemical parameters for group 1 | | | | | | |
|---|---|---|---|---|---|---|
| **Date** | **Biochemical parameters** | | | | | |
| | **AST** | **ALT** | **ALP** | **GGT** | **TP** | **Alb.** |
| **0 Day** | 141.7 | 68 | 168.7 | 65.1 | 3.1 | 2 |
| **7 Day** | 131.3 | 58 | 159.5 | 55.2 | 5.4 | 2.6 |
| **14 Day** | 106 | 46 | 141.6 | 44.5 | 6.3 | 3.2 |
| **21 Day** | 91.4 | 39 | 129.5 | 33.1 | 7.4 | 3.6 |
| The average value of biochemical parameters for group 2 | | | | | | |
| **Date** | **Biochemical parameters** | | | | | |
| | **AST** | **ALT** | **ALP** | **GGT** | **TP** | **Alb.** |
| **0 Day** | 143 | 58.8 | 168.3 | 74.5 | 2.3 | 2 |
| **7 Day** | 136.5 | 51.7 | 160.1 | 59.5 | 4.9 | 2.6 |
| **14 Day** | 124.5 | 44.1 | 146.3 | 42.7 | 6 | 3.3 |
| **21 Day** | 101.3 | 38.6 | 140 | 31.5 | 7 | 3.7 |
| The average value of biochemical parameters for group 3 | | | | | | |
| **Date** | **Biochemical parameters** | | | | | |
| | **AST** | **ALT** | **ALP** | **GGT** | **TP** | **Alb.** |
| **0 Day** | 150.7 | 61.6 | 172 | 62.7 | 3.1 | 2.1 |

| | | | | | | |
|---|---|---|---|---|---|---|
| **7 Day** | 140.6 | 53.8 | 164 | 53 | 4.6 | 2.4 |
| **14Day** | 130 | 46.5 | 156.7 | 45.3 | 5.7 | 2.6 |
| **21Day** | 117.4 | 42.7 | 151.5 | 40 | 6 | 2.8 |

Serum biochemical parameters reference range

| Biochemical Parameters | Unit | Sheep | Goat | Cow |
|---|---|---|---|---|
| AST | U/L | 49- 123 | 66- 230 | 60- 125 |
| ALT | U/L | 15- 44 | 15- 52 | 6.9- 35 |
| ALP | U/L | 27- 156 | 61- 283 | 18- 153 |
| GGT | U/L | 20- 44 | 20- 50 | 6- 17.4 |
| TP | g/dl | 5.9- 7.8 | 6.1-7.5 | 6.7-7.5 |
| Alb. | g/dl | 2.7- 3.7 | 2.3-4 | 2.5-3.8 |

Source ((Latimer, 2011) ALP= Alkaline phosphatase; GGT =gamma glutamyltransferase, AST = aspartate aminotransferase, ALT= alanine aminotransferase, ALP= alkaline phosphatas.
